# HPV16 Genomes: *In Silico* Analysis of E6 and E7 Oncoproteins in 20 South American Variants

**DOI:** 10.2174/0113892029293113240427065916

**Published:** 2024-05-09

**Authors:** Márcio Fabrício Falcão de Paula Filho, Lara Luísa Lopes Chrisóstomo, Isaac Farias Cansanção

**Affiliations:** 1 Medicine Collegiate, Campus Paulo Afonso, Universidade Federal do Vale do São Francisco (UNIVASF), Paulo Afonso, BA, 48605-780, Brazil

**Keywords:** HPV16, virus bioinformatics, viral diversity, evolution, E6 oncoprotein, software, cervical cancer

## Abstract

**Background:**

Human papillomavirus (HPV) is the main risk factor for the development of squamous cell cervical cancer, and E6 oncoprotein and E7 oncoprotein are important components of the viral genome and its oncogenic potential. It is known that different viral variants of HPV16 have different pathology and impact on the development of neoplasia, although few studies have been performed on South American variants.

**Objective:**

Therefore, the present study aimed to analyze *in silico* the genomic diversity of HPV16 in 20 complete genome variants of South America in the National Center for Biotechnology Information (NCBI) database.

**Methods:**

We performed a descriptive study to characterize the polymorphic regions of the E6 and E7 genes in HPV16 variants, using software for genomic data and single nucleotide polymorphism (SNP) analysis and others for phylogenetic analysis.

**Results:**

The variants analyzed included six SNPs linked to cancer (A131G, G145T, C335T, T350G, C712A, and T732C) and significant variation (798 nucleotide substitutions). Despite this, the variants showed low genetic diversity. Eighteen variants of unclear significance (VUS) were identified, 10 of which were in the coding E6 regions and 8 in the coding E7 regions. The prevalence of lineage D variants is of concern due to their pathology in cervical cancer and requires more research and epidemiological vigilance regarding their prevalence in the population.

**Conclusion:**

The data obtained in this study may contribute to future research on South American variants of HPV16, their pathogenicity, and the development of treatments.

## 1 INTRODUCTION

Human Papillomavirus (HPV) is one of the most important viruses transmitted in the population. Contamination is independent of penetrative sexual contact, and even if the transmission is sexual, it can manifest itself in other organs besides the genitourinary system. Today, 40 years after the discovery of the link between HPV and cervical cancer, it is still considered the most common way of developing this neoplasm [1-3]. It is also known to be responsible for the development of tumors in other sites, such as the vagina, penis, and skin [4]. To this end, further studies and an understanding of viral mechanisms have contributed to the development of preventive therapies and the introduction of testing protocols, such as the Pap smear, as well as the continuation of studies to understand the different types of viruses [3-7].

The WHO has set new goals to reduce the incidence of HPV in the world by 2030, such as mass testing and screening for women and vaccination of girls. According to the World Health Organization (WHO), there are estimated to be more than 600.000 new cases of cervical cancer discovered in 2020, among which 342.000 women died from the disease. In addition, the vast majority of cases occurred in underdeveloped countries, such as representatives from South America and Africa [7].

Even though it is known that HPV infection is worldwide spread and prevalent in over 95% of diagnosed cervical carcinoma cases [1, 2, 7-9], most of the epidemiological and genetic studies bring data only on European variants, leaving little knowledge of non-European variants [2].

Some viral variants for HPV represent a higher risk for the development of dysplasia when compared to others [10-12]. It is known that HPV16 and HPV18 are viral types with the highest risk of developing severe cervical cancer among HPV that affect humanity [1, 13]. Also, it is a consensus in biotechnology and pathology that the different viral variants for HPV16 have different pathologies and influences on the development of neoplasia, such as the course of the disease [1, 2, 9, 10, 14], reinforcing the need for more studies on existent and circulant viral variants.

An important constitutive element in HPV16’s viral particle and its pathogeny are the oncogenic protein (oncoprotein) E6 and E7, which were widely studied over their capacity in the development of neoplasias [1, 2, 10-13, 15-17]. It was found to be expressed in neoplasms, changing the expression of membrane factors in the cell cycle and depleting the host's immune response, facilitating the development of cancers associated with HPV and, overall, squamous cell carcinoma [1, 2, 10-13, 16, 17]. Thus, cervical cancer suppression can occur by inhibiting these genes, even in longer-lived stages [17, 18].

Together with the E6 protein, the E7 protein is a highly expressive oncoprotein that plays a role in carcinogenesis. Research indicates that even when patients receive extensive treatment, these proteins worsen their prognosis and increase their propensity to metastasize [4, 19]. In addition, other studies, including clinical phases in the vaccine manufacturing process, suggest that T cells have limited activity against E6 and E7 [6, 19]. Thus, the presence of the risk of new polymorphisms affects currently used therapies because they have not yet been described and, consequently, would help improve existing therapies and create new preventive agents [3, 6, 19].

Therefore, studies on HPV16’s E6 and E7 oncoproteins have shown great importance in terms of treatment, research, vigilance, and prevention for the world's population [3-6, 19]. This way, the present study aims at the genomic analysis, characterization, and compilation of genetic data from the oncogenes E6 and E7 in South America HPV16 variants as a way of helping to carry out more studies and indicate the improvement of prophylaxis, both drugs, and vaccination, for the region.

## 2 MATERIALS AND METHODS

We have made a descriptive study aiming to analyze the characterization of polymorphic regions within the genes E6 and E7 of HPV16. The collected data was secondary and obtained from public genome databases. The analyzed samples were obtained from the National Center for Biotechnology Information (NCBI), according to the following criteria: complete available genomes specific from South America and submitted up to the date of July 1st, 2023. This way, 20 genomes (KP212150.1, KP212151.1, KP212152.1, KP212153.1, KP212155.1, KP212157.1, KP874716.1, KP874717.1, KP874719.1, KU298881.1, KU298882.1, KU298883.1, KU298885.1, KP874718.1, HM057182.1, KP212154.1, KP212156.1, KP212158.1 and KP212159.1) were selected for analysis, having the reference genome for Human Papillomavirus type 16 (NC_001526.4) as a base for the study. After downloading, the sequences were aligned with the reference genome *via* MAFFT software. After selecting and processing the data, MEGA X and Ugene software were used to obtain genomic data of the 20 analyzed sequences. The obtained data for the study were: Length of the consensus sequence, Average sequence length; Number of analyzed sequences, Number of conserved sites; Variable sites; Number of singleton variable sites; Number of nucleotide substitutions, Synonymous mutations; Number of Parsimony informative sites; Number of uncertain bases; missing data/uncertain base; Transitions and Transversions.

Then, the HPV16 Genotyper [11] software was used to analyze the risk of Single Nucleotide Polymorphisms (SNPs) present in the variants and their impact on E6 and E7 oncoprotein. The obtained results on SNPs among the studied variants were compared to the available literature by ClinVar about these SNPs in humans to verify their pathogeny and the impact of those variants in populations. Also, we generated a cladogram for the phylogenetic analysis of the variants compared to the reference genome. For this, the utilized software was Iqtree v.2, with 1000 non-parametric bootstrap replications of robustness, and iTOL version 6.8.1 online (https://itol.embl.de) for visualization of the tree.

## 3 RESULTS

20 Genomic sequences were downloaded following the criteria: Complete genomes available at ClinVar database up to the date of July 1st, 2023. They were analyzed in comparison to the reference genome for Human Papillomavirus type 16 (NC_001526.4). As result for the structural and functional analysis of the genomes, Table **1** shows the values for sequence length ranged from 7842 (KP874718.1) to 7915 (HM057182.1), with an average of 7883 nucleotides among analyzed variants; 7652 conserved sites were found; 254 variable sites; 103 singleton variable sites; 798 nucleotide substitutions; 297 synonymous mutations; 597 non-synonymous mutations; 151 parsimony informative sites; 489 transitions and 309 transversions were identified in the present analysis.

Table **S1** shows a relation between variant genome, HPV16 Lineage, encoding region for E6 and E7 protein, and sequence length. revealing 14 genomes as Lineage A (KU298885.1, KU298883.1, KU298882.1, KU298881.1, KU298880.1, KP874719.1, KP874717.1, KP874716.1, KP212157.1, KP212155.1, KP212153.1, KP212152.1, KP212151.1, KP212150.1), 1 as Lineage B (HM057182.1), 1 as Lineage C (KP87418.1) and 4 as Lineage D (KP212154.1, KP212156.1, KP212158.1 and KP212159.1) (Table **S1**).

The query positions for E6 encoding were identified in a range from 50-526 (KP212155.1) to 7125-7601 (NC_001526.4). E6 coding region analysis revealed the length of 476 nucleotides for this gene. As for the E7 gene, its query positions ranged from 529-825 (KP212155.1) to 7604-7900 (NC_001526.4). The analysis revealed the length of 296 nucleotides. Table **S2** shows the identified SNPs for this study analysis, their nucleotide position, the variants in which they are present, the protein they are related to, and the impact of these polymorphisms in the referred protein (Table **S2**).

Table **S3** presents cancer-associated SNPs detected in the 20 analyzed genomes, detailing their nucleotide positions, variations, amino acid substitutions, present lineages, and their influence on the E6 and E7 proteins. Among these, three high-risk SNPs were identified: G145T (Q14H), C335T (H78Y), and T350G (L83V) (Table **S3**).

Finally, Fig. **S4** shows the cladogram for the studied variants. Lineage D variants were highlighted in red, Lineage C variant in orange, Lineage B in blue, and Lineage A in black (Supplementary material **S4**).

## 4 DISCUSSION

Previous studies on HPV16’s viral particle identified it as a possessor of low genetic variability [1, 2, 10]. However, the low variety in the genomes of variants might have a high impact on protein expression and pathogenesis of neoplasia [1, 2]. Thus, analysis of this variety shows its importance in understanding the evolutionary process of HPV16 and pathogenesis for its many variants.

The variant analysis of the present study, shown in Tables **1** and **S1**, identified genomic differences in the 20 analyzed South American variants compared to the reference genome (NC_001526.4). 14 genomes were identified as Lineage A (KU298885.1, KU298883.1, KU298882.1, KU298881.1, KU298880.1, KP874719.1, KP874717.1, KP874716.1, KP212157.1, KP212155.1, KP212153.1, KP212152.1, KP212151.1, KP212150.1); 1 as Lineage B (HM057182.1); 1 as Lineage C (KP87418.1) and 4 as Lineage D (KP212154.1, KP212156.1, KP212158.1 and KP212159.1).

The first observed difference among genomes was the sequence length, which ranged from 7842 nucleotides (KP874718.1) to 7915 nucleotides (HM057182.1), revealing additions and deletions in variant genomes when compared to the reference genome (sequence length: 7096), evidencing the evolutionary process of these variants [20].

The coding region for the E6 gene was different among variants and in the reference genome. In the reference genome, this region was comprehended between query positions 7125-7601. The positions for this region in variants ranged from 50-526 (KP212155.1) to 83-559 (KP212157.1; KU298880.1; KU298881.1; KU298882.1; KU298883.1; KU298885.1. In each of the analyzed variants and the reference genome, the length for the E6 gene was 476 nucleotides.

In the coding region for the E7 gene, the region defined by the consulted reference genome spans positions 7604-7900, while the position of the variants ranged from 529-825 (KP212155.1) to 562-858 (KP874719.1; KU298880.1; KU298881.1; KU298882.1; KU298883.1; KU298885.1), with 296 nucleotides analyzed.

When compared to the reference genome, the maintenance of the sequence length for the coding regions of E6 and E7 reveals no deletions or insertions in those genes along their evolutionary process. This way, the presence of polymorphisms evidences the evolutionary process that occurred in each variant while drifting from the reference genome [1, 20].

Besides, the analysis also evidenced the conservation of 7652 bases and 798 nucleotide substitutions, 249 synonymous mutations, and 549 non-synonymous mutations; 254 variable sites, among which 103 were singleton variable sites, representing a genetic variability of approximately 1.3% when comparing the 20 variant genomes to the reference genome for HPV16.

Mutations in nucleotide sequence might result in innumerous changes in gene expression and biological properties of the viral particle, such as protein binding, pathogenicity, tropism, host range, structural stability, replication, and resistance to environmental changes [1, 2, 21]. Thus, even though the viral particles of HPV16 and its variants have low genetic diversity when compared, the identified changes in the analyzed genomes might indicate significant changes in the replication capacity and pathogenesis of the viral particle [1, 2, 21].

Therefore, the absolute amount of nucleotide substitutions, non-synonymous mutations, and singleton variable sites found within this analysis confirms the presence of relevant genetic variability on HPV16’s South American variants and reveals a concerning scenario of changes in protein translation in these viral particles, evidencing the risk of their circulation and the need for more research in this area [2, 14, 21].

The E6 and E7 proteins regulate cellular checkpoints to establish cancer signaling. These proteins are, therefore, also referred to as “oncoplayers,” according to Pal and Kundu (2020), because they cause unchecked cell proliferation and angiogenesis, aid in the formation of metastases, and, because of the strong telomerase activity, encourage the viral genome's proliferation using the cellular machinery, rendering them oncogenic [17]. Some *in vitro* studies have shown that the absence of E6 and E7 leads to potentially carcinogenic cells undergoing apoptosis, underlining their importance. Furthermore, E7 was the first oncogene discovered in the HPV virus and helps interact with proteins that regulate the cell cycle and apoptosis by inhibiting the pRb protein. E6 is responsible for the inhibition of p53, leading to a loss of cell cycle regulation [17].

This way, table **S2** shows the analysis results on E6 and E7 SNPs found in the studied variants. A total of 26 SNPs were identified in the 20 variant genomes’ E6 and E7 coding region, among which seven are already described in literature over their roles in pathogenesis of squamous cell carcinoma (A131G, G145T, C335T, T350G, C712A, T732C), two are described in literature as silent mutations (T789C and T795G) and 17 were identified as Variants of Uncertain Significance (VUS) (A83C, T109C, G132C, C143G, T286A, A289G, A403G, A447G, G491A, A532G, T644C, A645G, A646C, A647C, T648C, G649T, A650T).

Table **S3** highlights the 7 cancer-related SNPs found in this study, 3 SNPs were identified in the present study, as well as their impact in E6 and E7 oncoproteins, the identified being identified as cancer-related SNPs: A131G, G145T, C335T, T350G, C712A, and T789C.

Compared to previous studies on SNPs, A131G, G145T, C335T, and T350G were identified in cases of Severe Cervical Dysplasia and head-and-neck carcinomas. Further examination into their pathology revealed that these SNPs can immortalize the E6 protein and enhance its effects, such as modulating the immune response of the host and the response of keratocyte’s membrane proteins [1, 2, 10, 12, 13, 16, 22].

C712A and T789C are cancer-related SNPs that affect the production of the E7 oncoprotein. The pathogenic effects of C712A are strictly related to the presence of A131G [21]; the presence of the combination of A131G and C712A leads to enhancement in the oncogenic potential of HPV [21]. Lastly, T732C is associated with low oncogenic potential [23].

For the present study, A131G was only identified in Lineage A variants, while C712A was only identified in lineage D variants. The pathogenesis of A131G is commonly associated with the presence of C712A, although it is not well established whether and how this interaction occurs, especially because C712A was not well studied over its pathogenicity [22].

Lineage A variants were identified as polymorphic for T350G, which means that variants in this lineage might manifest this SNP [1, 12]; Lineages B and C are monomorphic T in this nucleotide position, which means variants of these lineages cannot manifest this SNP; And Lineage D variants are monomorphic G in this nucleotide position, which means variants of this lineage always manifest this SNP [1]. G145T and C335T were only identified in Lineage D variants.

T350G, the polymorphism identified in Lineage D and A variants, is widely described in the literature as responsible for transformation in membrane proteins of keratocytes and changes in C33-A cell lines expression, leading to a higher risk of developing cervical cancer [2, 10, 12, 13]. According to Togtema *et al.* (2015) and Niccoli *et al.* (2012), the T350G SNP is directly associated with Severe Cervical Dysplasia. This polymorphism is related to the promotion of binding between oncoprotein E6 and several cellular proteins, such as P53 and apoptosis-inducing factors in C33-A cell lines, leading to the formation of complexes that inactivate important functions in these cells, such as tumor suppression, apoptosis, and regulation of mitosis, leading to tumor formation, epithelial degradation and, therefore, carcinomas [10-13, 15, 16].

G145T and C335T were identified as only functional in the presence of both SNPs [1, 13]. These SNPs are responsible for the capacity of transformation and immortalization of E6 through the degradation of apoptosis-inducing factors, such as p53 [1, 2, 16]. Thus, individuals infected by HPV16 lineage D variants might not respond to antiviral treatments and/or develop more severe forms of this condition due to the immortalization and capacity of transformation of E6 produced by these variants [16].

Since the pathology for A131G, G145T, C335T, and T350G is strictly related to the capacity of E6 and E7 oncoproteins to form complexes with cellular proteins, it is possible to confirm that these SNPs, as well as the oncoprotein E6, are not mutagenic.

The present study identified a total of 26 SNPs among the 20 complete genomes of HPV16 South America variants. Among those, 18 were identified as Variants of Uncertain Significance (VUS): A83C, T109C, G132C, C143G, T286A, A289G, A403G, A447G, G491A, A532G, G571A, T644C, A645G, A646C, A647C, T648C, G649T and A650T. These polymorphisms were identified in lineage B and D variants. No conclusive data about these polymorphisms could be found in the literature.

The phylogenetic analysis of the present study, shown in the cladogram in Supplementary material **S4**, corroborates previous findings in the literature on HPV16’s evolutionary analysis. It is shown that the analyzed genomes are splitting into sub-lineages over the timeline [1, 2, 20].

Lineage A and their sub-lineage variants were evolutionary closer to each other and the reference genome, showing low evolutionary pressure in their lifetime, while Lineage D variants and their sub-lineages were evolutionary distant from the reference genome and evolutionary closer to the other lineage D variants.

## CONCLUSION

The present study brought descriptive genetic data on South American variants for the E6 oncogene of HPV16 compared to the reference genome. *In silico* analysis of the obtained genomes resulted in phylogenetic data, statistical and genetic data on E6 oncoprotein, HPV16 E6 protein polymorphisms pathogenesis and the registration of Variants of Uncertain Significance. Through literature review and *in silico* analysis, we identified that Lineage D variants are possessors of a higher risk of developing severe cervical dysplasia. Few studies were found on descriptive data on South American variants and polymorphisms, which made it difficult to proceed with comparative analysis. This way, the present study brings important data for future research on non-European HPV16 variants and reinforces the need for more studies on South American variants, their genetics, and their pathology, which can lead to the improvement of previous treatments, such as vaccination, or the creation of new therapies for HPV in the region and, therefore, reduce the female mortality rate.

## AUTHORS' CONTRIBUTIONS

It is hereby acknowledged that all authors have accepted responsibility for the manuscript's content and consented to its submission. They have meticulously reviewed all results and unanimously approved the final version of the manuscript.

## Figures and Tables

**Table 1:** Data were obtained from comparative data between the 20 variants and the reference genome for HPV16.

**Polymorphisms**	**Total**
Consensus sequence length	8796
Average sequence length	7883
Nº of analyzed sequences	20
Nº of Conserved sites	7652
Variable sites	254
Nº of singleton variable sites	103
Nº of nucleotide substitutions	798
Synonymous mutations	249
Non-synonymous mutations	549
Nº of Parsimony informative sites	151
Nº of uncertain bases (msd+ub)	285
*msd-missing data / *ub-uncertain base	236 / 49
Transitions	489
Transversions	309

## Data Availability

The authors confirm that the data supporting the findings of this research are available within the article.

## LIST OF ABBREVIATIONS

HPV: Human Papillomavirus
NCBI: National Center for Biotechnology Information
SNP: Single Nucleotide Polymorphism
VUS: Variants of Unclear Significance
WHO: World Health Organization
