# Supplementary material for: HPV16 Genomes: In Silico Analysis of E6 and E7 Oncoproteins in 20 South American Variants
Source: Curr Genomics. 2024 May 9;25(4):316–21. doi: 10.2174/0113892029293113240427065916 (PMC11327806; doi:10.2174/0113892029293113240427065916)
Supplement: Supplementary file 1 [file CG-25-316_SD1.pdf]

Supplementary Material

HPV16 Genomes: *In Silico* Analysis of E6 and E7 Oncoproteins in 20 South American Variants

Márcio Fabrício Falcão de Paula Filho[1], Lara Luísa Lopes Chrisóstomo[1] and Isaac Farias Cansanção[1,*]

[1]Medicine Collegiate, Campus Paulo Afonso, Universidade Federal do Vale do São Francisco (UNIVASF), Paulo Afonso, BA, 48605-780, Brazil

Table S1. Relation between analyzed genomes, HPV16 Lineage, E6 encoding region, E7 encoding region, and sequence length.

Sequence	Lineage	E6 Encoding Region	E7 Encoding Region	Sequence Length
**NC_001526.4**	A	7125-7601	7604-7900	7906
**HM057182.1**	B	82-558	561-857	7915
**KP212150.1**	A	62-538	541-837	7854
**KP212151.1**	A	82-558	561-857	7901
**KP212152.1**	A	81-557	560-856	7901
**KP212153.1**	A	78-554	557-853	7894
**KP212154.1**	D	72-548	551-847	7891
**KP212155.1**	A	50-526	529-825	7872
**KP212156.1**	D	78-554	557-853	7900
**KP212157.1**	A	83-559	562-858	7900
**KP212158.1**	D	60-536	539-835	7849
**KP212159.1**	D	77-553	556-852	7896
**KP874716.1**	A	62-538	541-837	7845
**KP874717.1**	A	63-539	542-838	7847
**KP874718.1**	C	62-538	541-837	7842
**KP874719.1**	A	62-538	541-837	7845
**KU298880.1**	A	83-559	562-858	7906
**KU298881.1**	A	83-559	562-858	7906
**KU298882.1**	A	83-559	562-858	7863
**KU298883.1**	A	83-559	562-858	7904
**KU298885.1**	A	83-559	562-858	7906

Table S2. The identified SNPs, variant genome, nucleotide position, nucleotide variation, and their impact on squamous cell carcinoma in HPV16 infection.

-	-	Identified SNPs	-	-
**Nucleotide Position**	**Nucleotide Variation**	**Variant Genome(s)**	**Gene**	**Impact**
83	A83C	HM057182.1	E6	Uncertain
109	T109C	KP874718.1	E6	Uncertain
131	A131G	KP212155.1	E6	Increased oncogenic potential when associated with C712A and T350G
132	G132C	HM057182.1	E6	Uncertain
143	C143G	HM057182.1<br>KP874718.1	E6	Uncertain
145	G145T	HM057182.1<br>KP212154.1<br>KP212156.1<br>KP212158.1<br>KP212159.1<br>KP874718.1	E6	Immortalization and transformation of E6 protein
286	T286A	HM057182.1<br>KP212154.1<br>KP212156.1<br>KP212158.1<br>KP212159.1<br>KP874718.1	E6	Uncertain
289	A289G	HM057182.1<br>KP212154.1<br>KP212156.1<br>KP212158.1<br>KP212159.1<br>KP874718.1	E6	Uncertain
335	C335T	HM057182.1<br>KP212154.1<br>KP212156.1<br>KP212158.1<br>KP212159.1<br>KP874718.1	E6	Immortalization and transformation of E6 protein
350	T350G	KP212154.1<br>KP212156.1<br>KP212158.1<br>KP212159.1	E6	Changes in C33A cell expression
403	A403G	KP874718.1	E6	Uncertain
447	A447G	HM057182.1	E6	Uncertain
491	G491A	KP212155.1	E6	Uncertain
532	A532G	KP212154.1<br>KP212156.1<br>KP212158.1<br>KP212159.1	E6	Uncertain
571	G571A	KP212155.1	E7	Uncertain
644	T644C	HM057182.1	E7	Uncertain
645	A645G	HM057182.1	E7	Uncertain
646	A646C	HM057182.1	E7	Uncertain
647	A647C	HM057182.1	E7	Uncertain

648	T648C	HM057182.1	E7	Uncertain
649	G649T	HM057182.1	E7	Uncertain
650	A650T	HM057182.1	E7	Uncertain
712	C712A	KP212155.1	E7	Increased oncogenic potential when associat-ed with A131G and T350G
732	T732C	KP212154.1<br>KP212156.1<br>KP212158.1<br>KP212159.1	E7	Low oncogenic potential
789	T789C	HM057182.1<br>KP212154.1<br>KP212156.1<br>KP212158.1<br>KP212159.1<br>KP874718.1	E7	Silent Mutation
795	T795G	HM057182.1<br>KP212154.1<br>KP212156.1<br>KP212158.1<br>KP212159.1<br>KP874718.1	E7	Silent Mutation

Table S3. Identified Cancer-related SNPs, their nucleotide position, nucleotide variation, and lineages.

Nucleotide Position	Cancerous SNP	Query Nucleotide	Nucleotide Variation	Lineage	Impact
131	G	A	A131G	A	Enhances oncogenic potential when associated with C712A
145	T	G	G145T	D	Immortalization and transformation of E6 protein
335	T	C	C335T	D	Immortalization and transformation of E6 protein
350	G	T	T350G	A, D	Change in C33A cells expression
712	A	C	C712A	D	Enhances oncogenic potential when associated with A131G
732	C	T	T732C	D	Low Oncogenic Potential

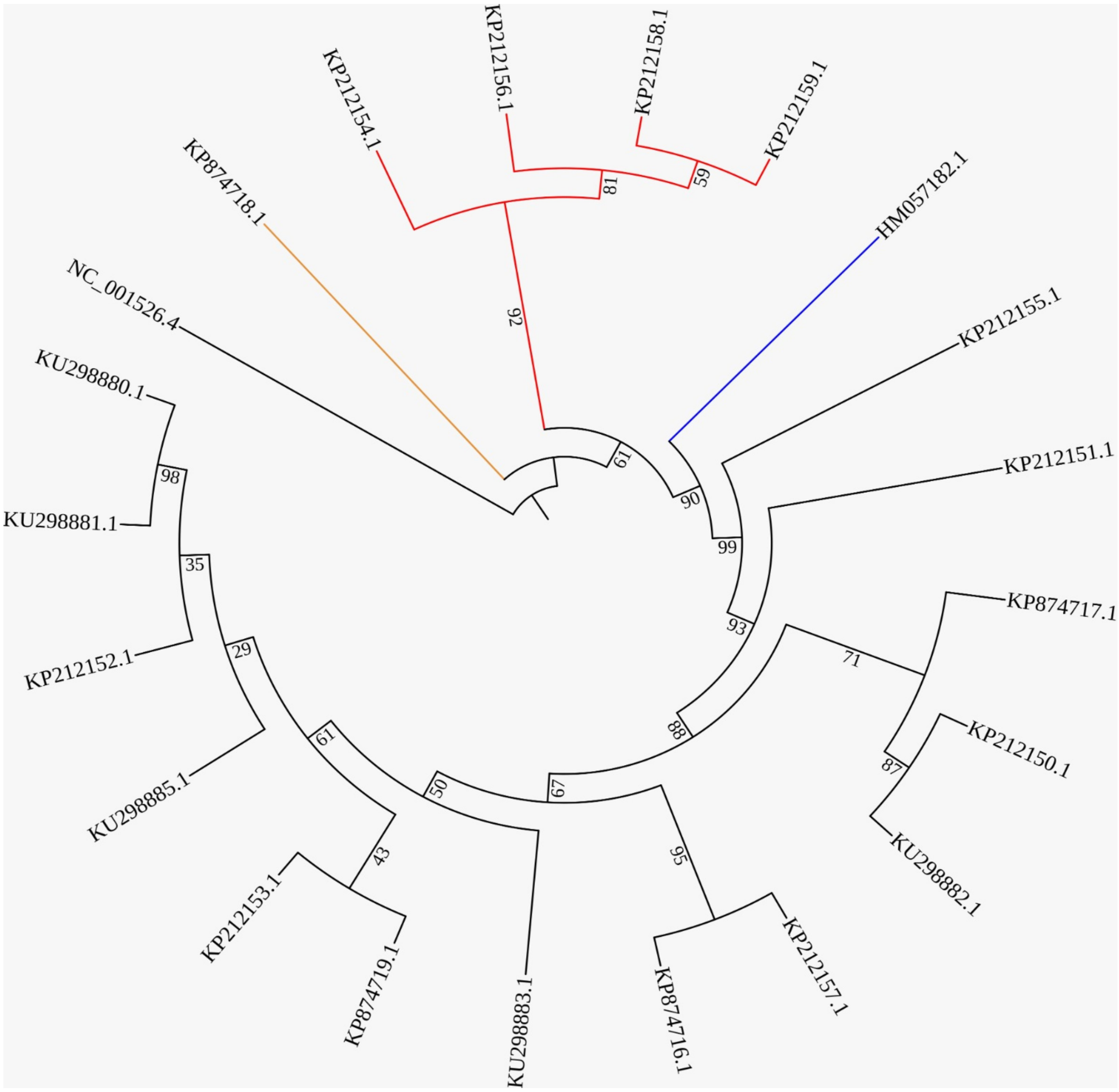

Fig. S1. Cladogram for phylogenetic analysis of the studied variants.
